# Determining the date of diagnosis – is it a simple matter? The impact of different approaches to dating diagnosis on estimates of delayed care for ovarian cancer in UK primary care

**DOI:** 10.1186/1471-2288-9-42

**Published:** 2009-06-23

**Authors:** A Rosemary Tate, Alexander GR Martin, Tarita Murray-Thomas, Sarah R Anderson, Jackie A Cassell

**Affiliations:** 1 Brighton and Sussex Medical School, Falmer, Brighton, BN1 9PH, UK; 2 Guy's and St. Thomas' NHS Foundation Trust, London, UK; 3 MHRA, GPRD, London, UK; 4 North West London Health Protection Unit, 61 Colindale Avenue, London, NW9 5EQ, UK

## Abstract

**Background:**

Studies of cancer incidence and early management will increasingly draw on routine electronic patient records. However, data may be incomplete or inaccurate. We developed a generalisable strategy for investigating presenting symptoms and delays in diagnosis using ovarian cancer as an example.

**Methods:**

The General Practice Research Database was used to investigate the time between first report of symptom and diagnosis of 344 women diagnosed with ovarian cancer between 01/06/2002 and 31/05/2008. Effects of possible inaccuracies in dating of diagnosis on the frequencies and timing of the most commonly reported symptoms were investigated using four increasingly inclusive definitions of first diagnosis/suspicion: 1. "Definite diagnosis" 2. "Ambiguous diagnosis" 3. "First treatment or complication suggesting pre-existing diagnosis", 4 "First relevant test or referral".

**Results:**

The most commonly coded symptoms before a definite diagnosis of ovarian cancer, were abdominal pain (41%), urogenital problems(25%), abdominal distension (24%), constipation/change in bowel habits (23%) with 70% of cases reporting at least one of these. The median time between first reporting each of these symptoms and diagnosis was 13, 21, 9.5 and 8.5 weeks respectively. 19% had a code for definitions 2 or 3 prior to definite diagnosis and 73% a code for 4. However, the proportion with symptoms and the delays were similar for all four definitions except 4, where the median delay was 8, 8, 3, 10 and 0 weeks respectively.

**Conclusion:**

Symptoms recorded in the General Practice Research Database are similar to those reported in the literature, although their frequency is lower than in studies based on self-report. Generalisable strategies for exploring the impact of recording practice on date of diagnosis in electronic patient records are recommended, and studies which date diagnoses in GP records need to present sensitivity analyses based on investigation, referral and diagnosis data. Free text information may be essential in obtaining accurate estimates of incidence, and for accurate dating of diagnoses.

## Background

Recent findings from three large international studies [1-3] suggest that the UK compares poorly with other countries in term of cancer survival. The reasons for this are not fully understood but may be due partly to delays in diagnosis and/or treatment after first onset of related symptoms, either because patients do not report their symptoms (patient delay), or because GP do not refer them quickly when they do (primary care delay). It is clear that a more thorough understanding of the extent and determinants of delay will be needed if cancer survival rates in the UK are to be improved [4], especially as the NHS cancer plan for England is now being questioned [5,6]. Mapping out routes from first symptom to diagnosis is currently the focus of much effort and is one of the main remits of a National Audit, within the National Awareness and Early Diagnosis initiative (NAEDI)) [7].

Although a number of studies have examined the different components of diagnostic delay in UK cancer patients, most notably the literature review and study by Allgar and Neal [8], many studies are based on small numbers and rely on patient interviews or surveys which may be subject to recall and non-response bias. In this study we investigate the potential and pitfalls of using records from a large UK primary care database, the General Practice Research Database (GPRD), for investigating such delays using ovarian cancer as the exemplar. Ovarian cancer was selected because diagnosis in its early stages greatly increases the probability of survival [9]. Although it is often thought of as an initially symptomless disease, there is increasing evidence that patients experience a number of symptoms, particularly abdominal and urogenitary symptoms before actual diagnosis [10-12].

In the UK, all residents are required to be registered with a General Practitioner who is the "gatekeeper" for specialist investigations and treatments undertaken in the National Health System. Thus, most women with this cancer will contact their general practitioner (GP) in the first instance [13,14], but there may be some delay between a patient first reporting to the GP and referral and diagnosis [13,15].

The GPRD [16] is one of the largest primary care databases in the UK. It contains anonymised longitudinal data on a representative sample of about 6% of the UK population – 3 million currently registered patients and over 8 million historic patients. The GPRD collects data from about 450 general practices throughout the UK and is widely used in research on disease epidemiology, drug safety and adverse drug reactions. Access to anonymised free text data is available in the database but at considerable additional cost. The main goals of the present study are to investigate the distribution of symptoms most commonly reported to the GP prior to ovarian cancer diagnosis, and to quantify the time between presentation of symptoms and diagnosis. Originally we planned to base our calculations on the first date that ovarian cancer was recorded (in common with previous studies of cancer symptoms using UK primary care records [17-20]). However, preliminary examination of individual records indicated that the first recorded code for ovarian cancer may not reliably indicate the date of diagnosis. Electronic patient recording in general practice allows flexible recording to take place and subsequently the completeness and accuracy of coded data is often variable [21]. In some instances, information may be stored only implicitly and it is not uncommon to find the actual diagnosis of a condition recorded at a late stage in the disease. In recognition of this fact, the main focus of the work reported in this paper is to explore alternative diagnostic dating methods for ovarian cancer using a number of working definitions to develop a generalisable strategy for analysis.

## Methods

### Data and measures used

#### Data

The GPRD dataset was provided under the MRC licence scheme and access to the dataset was approved by the Independent Scientific Advisory Committee (Protocol 07_069). The target population consisted of all females between 40 and 80 years of age who were alive and registered in the GPRD on June 1, 2002. From this population, all women with an incident diagnosis of ovarian cancer recorded during June 1, 2002 – May 31, 2007 were identified. Women with a previous definite or closely related diagnosis of ovarian cancer (Table 1) were excluded from the cohort. A medical diagnosis of ovarian cancer was defined by a Read or OXMIS code for this condition recorded in the patient's clinical or referral record i.e. Read codes: B440.00 (Malignant neoplasm of ovary) B440.11 (Cancer of ovary) or B44..00 (Malignant neoplasm of ovary and other uterine adnexa) or OXMIS codes: 1830A (Malignant neoplasm ovary), 1830AD (adenocarcinoma ovary), 1830C (Carcinoma ovary), 1830MC (mucinous cystadenocarcinoma ovary). Read codes (which have superseded the OXMIS codes) were specifically developed for use in UK primary care by Dr James Read during the 1980s are used to record all medical events in clinical practice. The Read code links alphanumeric labels to diseases and symptoms, allowing details of consultations to be entered and abstracted. Diagnostic codes start with a letter whereas symptoms, signs, investigations, procedures and administration tasks start with a number.

**Table 1:** READ codes for definite and very closely related diagnosis of ovarian cancer.

READ code	Name
	**Definite diagnosis**
B440.00	Malignant neoplasm of ovary
B440.11	Cancer of ovary
B44..00	Malignant neoplasm of ovary and other uterine adnexa
	**Very closely related diagnosis**
BB81.00	[M]Ovarian cystic, mucinous and serous neoplasms
BB81.11	[M]Ovarian cystadenoma or carcinoma
BB81.12	[M]Ovarian mucinous tumour
BB81.13	[M]Ovarian papillary tumour
BB81.14	[M]Ovarian serous tumour
BBC0.12	[M]Ovarian stromal tumour
B4...00	Malignant neoplasm of genitourinary organ
B4...11	Carcinoma of genitourinary organ
B553.00	Malignant neoplasm of pelvis
B553z00	Malignant neoplasm of pelvis NOS
B912.00	Neoplasm of uncertain behaviour of ovary
B913z00	Neoplasm of uncertain behaviour of female genital organs NOS
BB81200	[M]Serous cystadenocarcinoma, NOS
BB81800	[M]Papillary serous cystadenocarcinoma
BB81B00	[M]Serous surface papillary carcinoma
BB81E00	[M]Mucinous cystadenocarcinoma NOS
BB81H00	[M]Papillary mucinous cystadenocarcinoma
BB82.00	[M]Mucinous adenoma and adenocarcinoma
BB82100	[M]Mucinous adenocarcinoma
D212000	Anaemia in ovarian carcinoma

The definite diagnosis codes were used to select the cases for the study. The closely related diagnosis codes were use to exclude cases, who had a diagnosis prior to the study period.

Denominator data was provided to enable calculation of rates of a first definite diagnosis of ovarian cancer in the GPRD. This included information on patient count and number of person years stratified by calendar year, age, gender and practice.

From our dataset, 127 of the 414 practices were randomly selected to evaluate the current study objectives (data from the remaining practices will be used as validation data for testing the prognostic models which will be developed in the next stage of this work). The records from the 127 practices included 374 patients with a definite code for ovarian cancer. Of these 344 patients were used for this study after excluding 3 cases with a prior ambiguous diagnosis before the study period (1 "ovarian cystadenoma or carcinoma" and 2 "ovarian stromal tumour") and 27 patients who had been registered with the GP for less than 2 years before diagnosis.

#### Measures

The most commonly recorded ovarian cancer related symptoms were identified using a list of Read codes for commonly reported symptoms [12,22], drawn up with the help of a gynaecological oncologist (AM). Symptoms were subsequently categorized as follows. and grouped into the following 12 categories: 1. Abdominal pain, 2. Pelvic pain, 3. Back pain, 4. Abdominal distension/bloating, 5. Indigestion, 6. Nausea and Vomiting, 7. Constipation Change in bowel habits, 8. Urogenital Symptoms, 9. Abdominal mass, 10. Appetite Weight, 11. Tiredness, 12. Breathing problems. The percentage with symptoms and time between first relevant symptom and diagnosis code were assessed according to these categories.

Codes for relevant investigations and referrals for ovarian cancer were categorised into 5 groups: 1. Oophorectomy 2. Laparotomy or laparoscopy 3. Ultrasound 4. CA12 5. Referral to Gynaecologist. In order to pick up any codes for relevant symptoms, investigations or referrals which may have been missed, we examined the anonymised records of individual patients in the six-month period before definite diagnosis date, and also tabulated the most commonly occurring Read codes in order of frequency. The code list for the four categories listed in the section on sensitivity analysis below, and the category for 'cancer from other sites' was created by merging the clinical and referral records for the cases in the defined time periods with a comprehensive list of all cancer codes. The descriptions for the merged events were then inspected by the authors and assigned to the appropriate category.

The code lists used in this paper, including a list used to identify patients who had previously been diagnosed with, or treated for another type of, cancer are provided in the additional material (additional file 1, 2 and 3). The Stata progam file for creating the categories of relevant investigations and referrals is available as additional file 4.

### Data analysis

The number of cases diagnosed with ovarian cancer in each year in the study was calculated by dividing the number of first-time definite diagnoses codes for ovarian cancer by the corresponding person years for that year for the total study population. The rates were stratified by 5-year age bands and were compared with the "Registrations of cancer diagnosed in 2004, England" and "Registrations of cancer diagnosed in 2005, England" as reported by the Office of National Statistics (ONS) [23,24]. The incidence of major categories of commonly reported symptoms was estimated for each time period by dividing the number of patients reporting each symptom at least once in the given time period by the number of patients. Software: Data management was undertaken using MySql http://http:/www.mysql.com and statistical analyses were performed using Stata 10 ((Stata Corporation, Texas, USA). Hardware: Apple Mac Pro.

### Sensitivity of delay in relation to different definitions of index date

In order to determine the possible effects of inaccurate dating on the estimates of percentage of symptoms and delays, a sensitivity analysis was carried out using 4 alternative categories of Read codes indicating a diagnosis of, or investigation for ovarian cancer.

**Category 1. Definite diagnostic code only **Read codes for a case of ovarian cancer or malignant primary ovarian neoplasm as defined above ("definite diagnosis" in Table 1).

**Category 2. More general "ambiguous" code which could indicate diagnosis of ovarian cancer **This category included ambiguous but very closely related Read code indicating possible ovarian cancer ("very closely related diagnosis" in Table 1) together with at least one more general codes such as "Cancer", "Secondary neoplasm of other specified sites" and "Carcinomatosis"

**Category 3. Cancer treatment or referral code **All codes indicating a prior cancer diagnosis e.g "cancer care review", "chemotherapy", referral to oncologist.

**Category 4. Investigation or referral code for suspected ovarian cancer **This category included codes for a relevant investigation (e.g ultrasound scan, CA125 test), diagnostic procedure (e.g. oophorectomy) or referral to gynaecologist. This definition was included in order to identify when the GP was first recorded as taking action to investigate the ovarian cancer.

Four index dates based on these categories were constructed for each case, in order of increasing inclusivity, beyond the first Read code indicating a definite diagnosis of ovarian cancer.

**Date 1 **Earliest recorded date of definite diagnostic code (Category 1)

**Date 2 **Date 1, or, if present, the first date of an "ambiguous" code (Category 2) if this occurs prior to but within two years of Date 1. If another type/site of cancer has been diagnosed any time during the 4 years prior to the eventdate associated with a generic cancer code (e.g. "Cancer" or "Carcinomatosis" preceded by "Multiple myeloma") then it will be assumed that the code refers to this previous type of cancer and Date 1 will be used

**Date 3 **Date 2, or, if present, first date of code indicating GP already knew of a cancer diagnosis (Category 3) if this occurs prior to but within two years of Date 2. If another type/site of cancer has been diagnosed any time during the 4 years prior to the eventdate associated with such a code, then it will be assumed that the code refers to this previous type of cancer and Date 2 will be used – e.g Date 3 for a patient with "cancer care review" preceded by "cancer of the breast" will be Date 2.

**Date 4 **Date 3, or, if present, first date of a investigation or referral to a gynaecologist if this is earlier than but within 12 months of Date 3. N.B. this is slightly different and captures referral if present so while dates 1 to 3 are likely to be the same for most cases, 4 will generally be different as most will have had an investigation or referral code prior to a diagnosis.

## Results

### Number of women definite ovarian cancer codes in the entire GPRD

The total number of women aged between 40 and 80 at the beginning of the defined study period with a first unambiguous diagnosis of ovarian cancer recorded in the GPRD between June 1, 2002 – May 31, 2007 was 1166. All of these had Read (as opposed to OXMIS) codes. Table 2 shows the rate for each year. These rates were approximately 10% lower than the incidence rates reported by the Office of National Statistics (ONS) [23,24], for most age groups except for women aged between 75–80 years at diagnosis, when the the rates were approximately 30% lower.

**Table 2:** Number of episodes of a first recorded definite ovarian cancer code in the GPRD from 01/06/2002–01/06/2007 inclusive, among women aged between 40–80 at the start of this period

Year	Cases	Rate per 100,000 of first recorded code	Registered	Person years
2002	129	31.1	726432	414439
2003	220	31.1	743517	706756
2004	240	33.6	742736	713894
2005	241	33.8	747791	713711
2006	227	32.3	742409	702640
2007	109	37.9	705855	287784
Total	1166			

### Investigation of symptoms prior to definite diagnosis (Date 1) for the 344 selected patients

#### Incidence of symptoms

**Figure 1:**
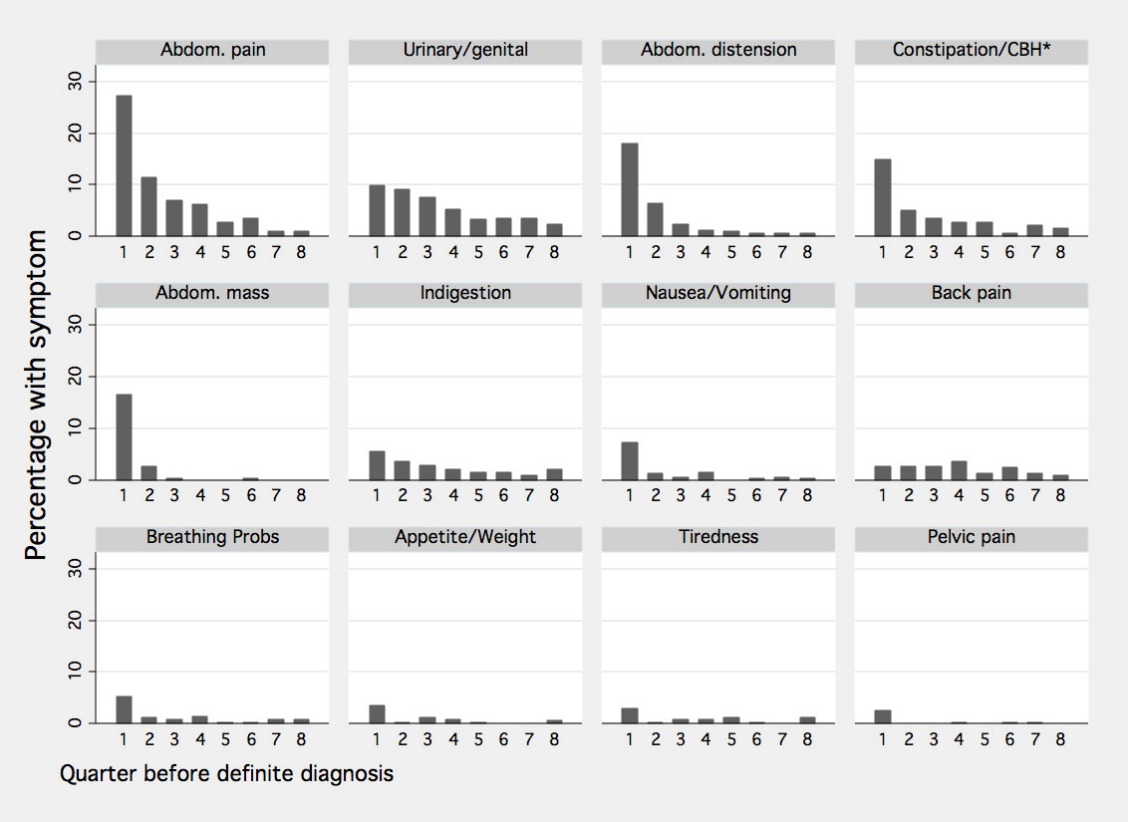
**Percentage of symptoms, reported at least once, by quarter before definite diagnosis**.

Three hundred cases (87%) were recorded as having had at least one of most commonly coded ovarian cancer related symptoms prior to definite diagnosis. The most commonly coded ovarian cancer related symptoms in the 12 months before diagnosis was abdominal pain (41%), followed by urogenital problems(25%), abdominal distension (24%), and constipation/change in bowel habits (23%), with 70% of cases reporting at least one of these. The percentage recorded as experiencing symptoms was much higher in the 3 months before diagnosis (69%) than in the preceding quarters (Figure 1). The other most commonly reported symptoms were cough (12%) and chest infection (6%).

#### Time between reporting symptoms and definite diagnosis

The median time in weeks between first recording a symptom and definite diagnosis was 19.5 for any symptom (n = 300), 13 for abdominal pain (n = 141), 21 for urogenital problems (n = 86), 9.5 for abdominal distension (n = 84) and 8.5 for constipation (n = 80).

#### Incidence of investigations

245 cases (71%) had at least one relevant investigation or a referral to a gynaecologist recorded in the 12 months up to and including the date of recorded diagnosis. The median [IQR] time in weeks between a investigation or referral and definite diagnosis was 7[3,14] for an oophorectomy (n = 85), 8 [2,15] for a laparotomy or laparoscopy (n = 53), 9[4,20] for an ultrasound (n = 105), 7.5 [3.5,15.5] for a CA125 test (n = 80) and 9 [4,21], for referral to gynaecologist (n = 143). In the previous year 4 patients were recorded as having had had an oophorectomy, 1 a laparotomy, 5 an ultrasound and 18 were referred to a gynaecologist. The total number of cases recorded as having a investigation or referral to a gynaecologist in the two years prior to diagnosis was 254(74%).

Since it seemed implausible that over 25% of cases had been diagnosed without a prior investigation or referral we checked whether these patients had a record of a letter from a consultant or specialist, which might have contained information on diagnostic tests. When cases who had no record of a investigation or referral, but who did have a Read code recording a letter (content unknown) from a consultant or specialist were included as having had a investigation or referral, the numbers increased to 285 (83%) in the previous year and 293 (85%) in the previous two years.

### Sensitivity of time of diagnosis to definition of diagnosis date

**Figure 2:**
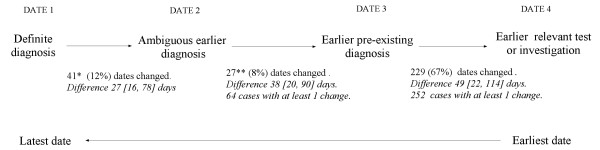
**Number of cases with index date change and median [IQR] difference between the earlier and later index date, according to the four definitions of index date**. Medians were calculated only for those with a index date change. *Date 2 for 6 cases with prior diagnosis of another cancer left unchanged. **Date 3 for 11 cases similarly left unchanged.

Preliminary examination of individual records indicated that first recorded code may not reliably indicate the date of diagnosis. For example, 4 cases had been coded as having an oophorectomy and 10 as an oncology or cancer care referral at least 13 weeks before the first diagnosis code. In addition, some cases had been coded with an "ambiguous" code some time prior to being given a definite diagnosis code. In 19% of cases (n = 64) the GP appeared to have already known that the patient had ovarian or a closely related cancer prior to recording a definite diagnosis (Figure 2). Of these, 47 cases had a prior ambiguous diagnosis, including 1 who had a diagnosis of "Carcinoma in situ of ovary" 31 months prior to the definite diagnosis. This patient was kept in the study since the diagnosis was within the prescribed study period. Twenty-six patients, who had no record of a prior cancer diagnosis, had received cancer treatment or had been referred to an oncologist prior to an "ambiguous" diagnosis. These included 10 cases coded as "Seen in oncology clinic", 3 as "Cancer care review" and 3 as "Chemotherapy" over 4 weeks before an "ambiguous" diagnosis. Four cases had been coded as having an oophorectomy and 10 as an oncology or cancer care referral at least 13 weeks before the first diagnosis code. The median differences between a previous and subsequent index date, for those whose index dates were changed, were all within two months of the previously defined index date, although in some cases the differences were much greater (as shown by the IQR). The median difference between Date 1 and Date 4, for the 229 cases who had a prior code for a investigation or referral for ovarian cancer was 8 [3,19] weeks.

**Figure 3:**
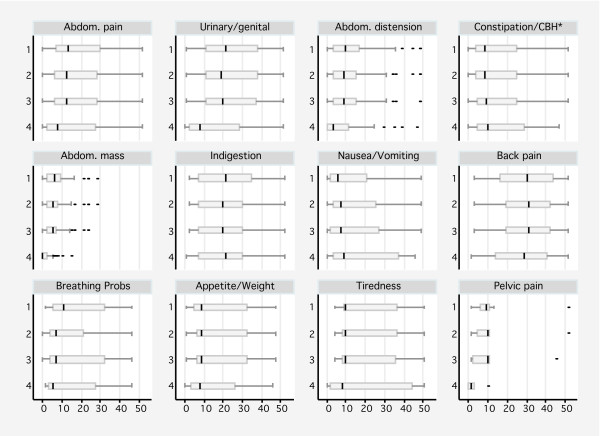
**Box plots showing the distribution of time (weeks) between a symptom being first reported and a diagnosis according to the four definitions: Date 1**. definite diagnosis, Date 2. diagnostic code plus closely related codes, Date 3. diagnosis code to include complication or treatment codes and Date 4. diagnostic code modified by investigation or referral code. Symptoms are ordered by frequency of occurrence

For most of the ovarian cancer symptoms, the percentage of cases recorded as having the symptom in the year prior to diagnosis changed only slightly with each subsequent definition of index date (Table 3), except for between Date 3 and 4 when the percentages decreased for all the most commonly reported symptoms. The time between recording one of the 4 most common symptom and diagnosis changed very little for Dates 1 to 3 ((Table 3 and Figure 3). However the time between recording a symptoms and first investigation/referral was considerably shorter for Date 4 than Dates 1–3 for most of these symptoms, with the exception of constipation/change in bowel habits where the time increased from 8.5 to 10 weeks (probably due to the proportion being smaller before referral than diagnosis).

**Table 3:** Percentage reporting a symptom and (in italics) the median time in weeks between first report of symptom and date of diagnosis or investigation/referral in the year before each index date

Symptom	Date1		Date2		Date3		Date4	
	%	median delay (weeks)	%	delay	%	delay	%	delay
Abdominal pain	**41**	*13*	**41**	*12*	**41**	*12*	**38**	*8*
Urogenital	**25**	*21*	**25**	*19*	**25**	*19.5*	**24**	*8*
Abdominal distension	**24**	*9.5*	**24**	*9*	**24**	*9*	**20**	*3*
Constipation/change bowel habits	**23**	*8.5*	**23**	*8*	**23**	*9*	**19**	*10*
Abdominal mass	**19**	*6*	**20**	*5.5*	**19**	*5*	**15**	*0*
Indigestion	**11**	*21*	**11**	*19.5*	**11**	*20*	**9**	*21*
Nausea/Vomiting	**10**	*6*	**8**	*7*	**8**	*7*	**6**	*9*
Back pain	**9**	*30.5*	**10**	*31*	**10**	*31*	**9**	*28.5*
Breathing Probs	**8**	*11*	**8**	*7*	**8**	*7*	**7**	*5.5*
Appetite/Weight	**6**	*9*	**5**	*9*	**5**	*9*	**4**	*8*
Tiredness	**5**	*10*	**5**	*9.5*	**5**	*10*	**6**	*8*
Pelvic pain	**3**	*9*	**3**	*10*	**3**	*10*	**2**	*1.5*

Any one of these symptoms	**87**	*19.5*	**87**	*18*	**87**	*18*	**85**	*10*

## Discussion

A high proportion of the 344 patients with ovarian cancer were recorded as reporting abdominal, gastrointestinal or urogenital symptoms to their GP in the 6 months before a definitely recorded diagnosis (Date 1) with half of these being diagnosed within four months of first recorded symptom. Our study confirms, using contemporaneous observational data, that a substantial number of patients do indeed consult with relevant symptoms prior to first referral by the GP. The proportion of ovarian cancer patients recorded as experiencing ovarian cancer symptoms is similar to that seen of other studies based on retrospective analysis of patient records [13,25-28]. However it is lower than those based on self-report, indicating that symptoms may either be under-reported, or under-coded in patient records.

For half the cases, the time from first symptom report to first referral or investigation was 8 weeks or less. These findings contrast with those of Kirwan et al's [13] retrospective study of GP patient notes, which found that 50% of cases were referred to hospital directly after first consulting their GP on ovarian cancer related symptoms. However, since their estimates of delay were shorter than those reported by studies that have examined primary care delay in other cancers [8], their findings may have been overly optimistic. The work described in this paper is the first part of a study aiming to develop and test prognostic models based on symptoms and consulting patterns. We present a generalisable strategy for investigating inaccuracies in dating of diagnosis and their effect on estimates of symptoms and delay in UK primary care databases. We suggest that this or a similar explicitly stated strategy should always be followed for studies which require the dating of symptoms in relation to diagnosis, and that sensitivity analyses should be undertaken for definitions of diagnosis date. Although using different index dates based on diagnosis made little difference to the percentages of recorded symptoms and estimates of delay in this particular study, this may not always be the case depending on the disease. We also recommend that, for studies investigating primary care delay, the date of first investigation for suspected disease, rather than the diagnosis date as the index date, is much more relevant. Using the date of diagnosis as index date to investigate " red flag" symptoms (as was done for example in [17]) may be misleading since the actual diagnosis will be made in the hospital, and will usually be recorded later by the GP.

An advantage of using electronic patient record databases for epidemiological research is that they contain information on large and representative numbers of patients that is recorded during consultation, and therefore studies based on these data are much less prone to recall or nonresponse bias. However, the use of data that has been recorded for administrative reasons, rather than for research, is associated with a different set of problems; some information may be missing or incomplete, or possibly only recorded in the (less accessible) free text notes. It is clear that even for a major disease such as ovarian cancer not all events are (or can be) coded at the time of definitive diagnosis or even at all. The lower incidence of ovarian cancer codes in the GPRD records compared with the ONS figures provides evidence of this under-recording and concurs with a recent study of patients from 5 UK general practices [25] which found that 20% of cancer cases reported in the cancer registry could not be identified as such in the GP records. In this study we looked only at individual symptoms, rather than combinations, and have no detailed analyses of temporal information on sequences of events. Primary care records only allow us to investigate primary care delay and we acknowledge that there is likely to have been an under-recording of symptoms, either because the patient did not report them, or because the GP did not code all the symptoms reported by the patient. We have to date analysed only coded GPRD data and have not examined the free text part of the records which may contain further information on diagnosis (e.g. in the hospital letters) and are likely to also contain important information on the severity of symptoms or on additional symptoms which have not been coded.

## Conclusion

If epidemiological and health services research based on electronic records is to be of maximal public health benefit, it will be important to develop methodologies for the understanding and appropriately anonymised extraction and use of information "concealed" within the free text. Studies of the incidence of serious illness, and of survival and patterns of care, will increasingly draw on the analysis of routine health service records, which are not primarily designed for research or audit, but to assist clinicians in caring for their patients. It is therefore vital that strategies are developed by which the impact of variation in clinician recording patterns on epidemiological estimates can be better understood, compared and adjusted for across the spectrum of disease.

## Competing interests

The authors declare that they have no competing interests.

## Authors' contributions

ART conceived and wrote the paper, and carried out all the analyses. JC initiated and coordinated the study, and was involved in writing the paper. AM drew up the original code lists, provided expert clinical advice and contributed to the writing of the paper. TMT assisted with the design of the study, provided the GPRD data set, and contributed to the writing of the paper.

## Pre-publication history

The pre-publication history for this paper can be accessed here:

http://www.biomedcentral.com/1471-2288/9/42/prepub

## Supplementary Material

Additional file 1 **Codelist for the four categories described in the section on sensitivity analysis**. List of read codes and categories that were used for defining dates 1–4. Click here for file

Additional file 2 **Codelist for ovarian cancer symptoms**. List of read codes and categories used in this paper to calculate the incidence of symptoms. Click here for file

Additional file 3 **Codelist for all types of cancers**. The codelist that was used to identify prior cancers in other sites. Click here for file

Additional file 4 **Stata program for creating categories for investigations and referrals**. The "do" file that was used for creating these categories. Click here for file
